# Detection of *Brucella abortus* Vaccine Strain RB51 in Water Buffalo (*Bubalus bubalis*) Milk

**DOI:** 10.3390/pathogens11070748

**Published:** 2022-06-30

**Authors:** Daniela Averaimo, Fabrizio De Massis, Giovanni Savini, Giuliano Garofolo, Flavio Sacchini, Anna Abass, Manuela Tittarelli, Giacomo Migliorati, Antonio Petrini

**Affiliations:** National and WOAH Reference Laboratory for Brucellosis, Istituto Zooprofilattico Sperimentale dell’Abruzzo e Molise “G. Caporale”, Campo Boario, 64100 Teramo, Italy; d.averaimo@izs.it (D.A.); g.savini@izs.it (G.S.); g.garofolo@izs.it (G.G.); f.sacchini@izs.it (F.S.); a.abass@izs.it (A.A.); m.tittarelli@izs.it (M.T.); g.migliorati@izs.it (G.M.); a.petrini@izs.it (A.P.)

**Keywords:** brucellosis, vaccine, Rb51, water buffalo, milk

## Abstract

The isolation of *B. abortus* RB51 vaccine strain from a milk sample in a water buffalo farm in southern Italy emphasizes the risk to public health of consuming contaminated milk or milk products following illegal vaccination.

## Research Letter

*Brucellosis* is one of the most important zoonoses affecting livestock and humans worldwide [1,2]. *Brucella abortus* RB51 is a rough mutant strain derived from the virulent strain *B. abortus* 2308 after several passages on trypticase soy agar with rifampin [3,4]. It has been used as a vaccine for many years. In Italy, a brucellosis eradication program was implemented beginning in 1994 [5]. Considering the nature of the disease (vaccination cannot prevent the infection of the animal and the related carrier state), the passage from a brucellosis control program to a brucellosis eradication program would necessarily imply a ban on any kind of vaccination [6]. However, due to the particular epidemiological situation, vaccination was authorised in 6 to 9-month-old water buffalo in the province of Caserta, southern Italy, between 2003 and 2014. After the prevalence of brucellosis had fallen to acceptable levels, the vaccination ban was then reinstated. Nonetheless, in areas with relatively high prevalence, RB51 might be illegally used by farmers to reduce the number of abortions and the other economic losses associated with brucellosis, such as the drop in fertility and/or in milk production. [6]. Currently, the action plan implemented by the competent authorities to combat illegal vaccination of buffalo involves serological surveillance using a specific Complement Fixation Test with RB51 antigen (CFT-RB51) and bacteriological surveillance in milk.

It is common knowledge that cows vaccinated as adults may shed RB51 in milk [7,8,9]. Conversely, in water buffalo (*Bubalus bubalis*), RB51 shedding in milk has been observed in animals only under experimental conditions during the first week after a triple dose injection [10]. RB51 has zoonotic potential. Clinical cases of human brucellosis due to RB51 infection have been regularly observed following the consumption of raw milk or after occupational exposure [11,12,13,14].

This report describes the field isolation of *B. abortus* RB51 vaccine strain from a milk sample collected from a water buffalo.

In May 2021, the Italian Reference Laboratory for Brucellosis was commissioned by the competent authorities to confirm the supposed illegal use of RB51 on a buffalo farm in the province of Caserta. An initial screening was carried out on 809 serum samples using the RB51 specific complement fixations test (RB51-CFT), which was first developed and validated for cattle and later applied to buffaloes [15,16,17,18]. One hundred sixty animals tested positive for RB51-CFT (19.7%), with antibody titers ranging from 1:4 (the test cutoff) to ≥ 1:128. In a second round of sampling, 57 individual milk samples were collected from RB51-CFT seropositive buffaloes in lactation phase. The procedures for *Brucella* isolation described by the World Organization for Animal Health (WOAH) were performed [19], including weekly subcultures from *Brucella* broth base supplemented with polymyxin B sulphate (5000 units), bacitracin (25,000 units), natamycin (50 mg), nalidixic acid (5 mg), nystatin (100,000 units), vancomycin (20 mg), and amphotericin B (1 mg) per liter [20] onto solid media (Farrell and CITA). After five weeks of weekly subcultures, *Brucella* spp. strain was isolated in a milk sample from a buffalo born on the farm in question in May of 2018, that is, during a period in which vaccination was not allowed. This animal had a high RB51-CFT antibody titer value (≥1:128). The suspected isolate was assigned to the *B. abortus* vaccine strain RB51 by means of PCR-based tests, identifying strain-specific *wboA* gene disruption by an IS711 element [21]. A cgMLST analysis based on whole-genome sequencing confirmed the classification, showing that the strain allelic profile had only two allelic differences compared to the RB51 reference genome (GenBank accession number GCF_011801185.1). All raw reads generated were submitted to the National Center for Biotechnology Information (NCBI) under the accession number PRJNA804372.

Even though it was unclear when the animal was injected or what dose was used, finding the RB51 strain in buffalo milk is a result that can have significant health implications for both for consumers and farm workers.

*B. abortus* RB51 can be responsible for serious disease in humans, as the strain is naturally resistant to rifampin, one of the treatments of choice for those patients that cannot be treated with tetracyclines or streptomycin (pregnant women, children, and cases of brucellar endocarditis and neurobrucellosis) [13]. Furthermore, being a rough strain, it does not elicit an antibody response detectable by the conventional serological tests for brucellosis [15,16,17,18,19,20,21,22,23].

The use of this vaccine strain outside official control may affect the spread of the field strain in a way that the effects on the control campaign cannot be properly assessed by the competent authority, thus potentially causing the failure of the control program towards eradication.

It is not easy to isolate *Brucella* spp. from milk samples due to the intermittency of shedding [24,25] and the possibility of shedding at low levels [26] in the case of vaccinated animals, as previously reported in [14]. In these situations, WOAH procedures suggest the use of liquid enrichment in order to improve the sensitivity of the isolation test.

In our report, despite the relatively high number of animals that tested positive for RB51-CFT, *Brucella* spp. was isolated in only one animal, and only after five weeks of incubation. The long period could be due to the use of Farrell’s medium, which has been shown to inhibit the growth of RB51 [4]. Even if in this case report, *Brucella* was isolated only in Farrell’s medium, which was due to high presence of contaminating microorganisms; the use of other specific media, such as CITA, is recommended for the isolation of RB51 [27]. Furthermore, this would justify the low degree of isolation of RB51 in the field, and may be a reason for the elimination of RB51 from vaccinated animals as adults being underestimated. To the best of our knowledge, this is the first report of RB51 isolation from water buffalo milk under field conditions. This confirms that illegal use of *B. abortus* RB51 vaccine in this species might represent a serious threat to public health.
